# An observational study of quick-relief inhaler storage and use among children with asthma in home, school, and community

**DOI:** 10.3389/fped.2026.1797219

**Published:** 2026-06-01

**Authors:** Caroline Luff, Devika Jaishankar, Uma Balachandran, Alexandra Knitter, Anna Volerman

**Affiliations:** University of Chicago, Chicago, IL, United States

**Keywords:** adolescents, asthma, children, inhaler, self-management

## Abstract

**Background:**

For children with asthma, access to and use of quick-relief inhalers can be life-saving. Children must have access and ability to use inhalers across home, school, and community settings. This study aimed to examine how children store and use inhalers across settings and evaluated the relationship to asthma control and exacerbations.

**Methods:**

This cross-sectional study recruited participants 10–17 years with asthma from clinics affiliated with one urban academic medical center. Children completed a questionnaire with questions about quick-relief inhaler storage and use within home, school, and community settings as well as asthma control and exacerbations. Responses were categorized by whether storage or use were independent or not independent. Kendall's Tau was calculated to assess correlation between children's independence of inhaler storage and use across settings. Chi-squared and Fisher's exact tests examined their association with asthma control and exacerbations.

**Results:**

Among 90 enrolled children, 53% (*N* = 48) were male and 67% (*N* = 60) were non-Hispanic Black. Mean age was 13.1 years (SD = 2.2). At home, 52% (*N* = 47) of children reported independently storing quick-relief inhalers and 54% (*N* = 48) independently used inhalers. At school, 60% (*N* = 54) of children indicated they independently stored inhalers while 82% (*N* = 74) independently used inhalers. In the community, 90% (*N* = 81) of children reported independently storing inhalers. Within settings, there was moderate correlation between independent inhaler storage and use. Across settings, independent inhaler use was moderately correlated between home and school; however, independent storage of inhalers was not correlated. Also, inhaler storage and use was not associated with asthma control or exacerbations.

**Conclusions:**

This observational study is among the first to describe quick-relief inhaler storage and use by children across settings, showing variation in practices. Although we found storing and using quick-relief inhalers independently were correlated at home and school, they were not associated with asthma control or exacerbations. These findings help identify patterns related to asthma self-management and highlight opportunities for collaboration between clinicians, caregivers, and children to support developmentally-appropriate asthma care across settings.

## Introduction

For the 4.7 million children with asthma, poor disease control can impede daily activities and lead to emergency situations (1). Given the potential life-saving benefits of quick-relief inhalers, it is critical that inhalers are readily accessible to support effective asthma self-management.

One key component of self-management is the independent carry and use of inhalers (self-carry/use). This practice is critical to ensure youth have quick access to their inhalers in case of asthma symptoms, particularly among older children and adolescents as they move between different settings (e.g., home, school). For instance, prior research indicates children who are allowed to independently carry their inhalers at school have greater confidence in preventing asthma exacerbations (2). Such confidence in preventing exacerbations, known as self-efficacy, is associated with improved quality of life for individuals with asthma (3).

While it is recognized self-carry and use of inhalers are important for asthma care, little is known about such practices among youth and how they relate to asthma control (3). This study examined how children store and use inhalers across different settings and evaluated how they are related to asthma control and exacerbations.

## Methods

This cross-sectional study utilized baseline data from a randomized controlled trial of inhaler education strategies conducted in 2020–2021. The study was approved by the University of Chicago Biological Sciences Division Institutional Review Board (IRB19-2091), based on US Federal Policy for the Protection of Human Subjects. The study is reported in alignment with STROBE guidelines for reporting observational studies.

Participants were children 10–17 years with physician-diagnosed asthma and their caregivers. Recruitment occurred in clinics affiliated with one urban academic medical center in Chicago. Informed consent was obtained prior to inclusion in the study.

At the initial study visit, children independently completed a questionnaire, including sections on inhaler storage/use and asthma control. Quick-relief inhaler storage/use were assessed for home, school, and community settings using free-response questions. For each setting, the child reported where they store and who helps them administer their quick-relief inhaler. These responses were each categorized as binary (independent or not independent). Inhaler storage was categorized as “independent” if the child reported their inhaler is stored in spaces primarily accessible to them (e.g., bedroom, backpack, pocket) versus “not independent” if it is stored in places mainly accessible to adults (e.g., kitchen, office, parent's car). Inhaler use was categorized as “independent” if the child reported administering the inhaler on their own versus “not independent” if they required assistance from another individual. For the community setting, data was collected on inhaler storage but not use. At the same study visit, asthma control was assessed with the validated Childhood Asthma Control Test (CACT) (10–11 years) or Asthma Control Test (ACT) (12–17 years) (well-controlled asthma: score > 19). Asthma exacerbations were measured by parent report of the child requiring oral steroids, having emergency department visit, or having hospitalization related to asthma in the past 12 months.

For analysis, descriptive statistics were calculated. Chi-squared tests were used to examine differences in inhaler storage and use across settings based on age groups (10–13 years versus 14–17 years). Kendall's Tau correlation coefficient evaluated the association between children's independence of inhaler storage and use across settings. Chi-squared and Fisher's exact tests examined whether there was a relationship between asthma control and exacerbations with inhaler storage and use. Significance was defined by two-tailed *p* < 0.05. Analysis was conducted using Radiant (R package version 1.6.0).

## Results

Ninety children and their parents enrolled and completed the baseline visit between August 2020 and August 2021 (Table 1). Children's mean age was 13.1 years (SD = 2.2). Participants were 53% male (*N* = 48) and 67% non-Hispanic Black (*N* = 60). Most children had well-controlled asthma (75%, *N* = 67) and good self-management (73%, *N* = 66). Over half of (56%, *N* = 50) children had an exacerbation in the last 12 months, including 52% (*N* = 47) requiring oral steroids, 36% (*N* = 32) having an emergency department visit, and 16% (*N* = 14) having a hospitalization for asthma.

**Table 1:** Demographics and asthma characteristics of participating children (*N* = 90).

Characteristic		*n* (%)
Age, mean (SD)		13 (2.2)
Gender	Female	42 (47)
Race	Non-Hispanic Black	60 (67)
	Hispanic	13 (14)
	Non-Hispanic - White	12 (13)
	Non-Hispanic Other or multiple races	5.0 (5.6)
Asthma control^a^	Well-controlled	67 (75)
	Poorly controlled	23 (25)
Asthma exacerbations^b^	Any	50 (56)
	Oral steroids	47 (52)
	Emergency department visit	32 (35)
	Hospitalization	14 (16)
Independent inhaler storage^c^	Home	47 (52)
	School	54 (60)
Independent inhaler use^c^	Home	48 (53)
	School	74 (82)

a Asthma control was measured using asthma control test (ACT) and childhood asthma control test (cACT), based on age. Well-controlled asthma was defined based on score > 19, in alignment with ACT and cACT scoring criteria.

b Asthma exacerbations were measured by parent report of the child requiring oral steroids, having emergency department visit, or having hospitalization related to asthma in the past 12 months.

c Inhaler storage and use at home and school were categorized into two groups: “independent” and “not independent.” Use was deemed independent if the child reported administering the inhaler on their own and “not independent” if the child required assistance from another individual. If the child indicated an adult assists with medication administration, a follow-up question asked about the extent of adult involvement (i.e., they watch me use my inhaler; they talk me through using my inhaler; they do some of the steps for me to use my inhaler; they do all of the steps for me to use my inhaler).

At home, half the children reported independently storing (52%, *N* = 47) and using (53%, *N* = 48) quick-relief inhalers. For example, children who independently stored their inhalers reported they were “in my room in dresser” and “purse/closet”, while children with not independent storage indicated the inhaler was store in “mom and dad's room”, “my grandma's closet”, and “with mom”. Meanwhile, at school, most children indicated they independently stored (60%, *N* = 54) and used (82%, *N* = 74) these inhalers. For example, children with independent storage at school stated that their inhaler was kept in “my pocket” and “purse or backpack”, while those with not independent storage reported the inhaler was in the “school office”, “nurse's office”, and “with teacher”. In the community, 90% (*N* = 81) of children reported independently storing these inhalers. Also, when examined by age (Table 2), there were no differences in independent storage or use of inhalers by children when at home. A greater proportion of older children reported independent storage at school than younger children (76% vs. 47%, *p* = 0.0006), however there were no difference in independent use at school (83% vs. 82%, *p* = 0.87).

**Table 2:** Inhaler storage and use across settings as reported by children, by age group (*N* = 90).

Setting	Independent inhaler storage/use		*p*-value^a^
	10–13 years (*n* = 49), *n* (%)	14–17 years (*n* = 41), *n* (%)	
Home storage	27 (55)	20 (49)	0.55
Home use	23 (47)	25 (61)	0.18
School storage	23 (47)	31 (76)	0.006
School use	40 (82)	34 (83)	0.87

a Chi-squared test

Within each setting, there was a moderate correlation between a child's independence in quick-relief inhaler storage and use–both at home (Tau = 0.26, *p* = 0.01) and at school (Tau = 0.29, *p* = 0.009). Across settings, independent storage of inhalers was not correlated (home/school: Tau = −0.009, *p* = 0.9; home/community: Tau = 0.05, *p* = 0.6). However, independent inhaler use was moderately correlated across home and school (Tau = 0.38, *p* = 0.0003).

There were no significant relationships between asthma control or exacerbations with inhaler storage or use in both home and school (Table 3).

**Table 3:** Association between independence in inhaler storage/use across settings and asthma control (*N* = 90).

Outcome/setting	Independent *n* (%)	Not independent *n* (%)	Independent *n* (%)	Not independent *n* (%)	*p*-value^a^
Asthma control	Well controlled asthma (*n* = 67)		Poorly controlled asthma (*n* = 23)		
Home storage	33 (49)	34 (51)	14 (61)	9 (39)	0.34
Home use	36 (54)	31 (46)	12 (52)	11 (48)	0.9
School storage	39 (58)	28 (42)	15 (65)	8 (35)	0.55
School use	58 (87)	9 (13)	16 (70)	7 (30)	0.11
Asthma exacerbations^b^	No asthma exacerbations (*n* = 40)		Asthma exacerbation (*n* = 50)		
Home storage	24 (60)	16 (40)	23 (46)	27 (54)	0.19
Home use	25 (63)	15 (38)	23 (46)	27 (54)	0.12
School storage	22 (55)	18 (45)	32 (64)	18 (36)	0.39
School use	34 (85)	6 (15)	40 (80)	10 (20)	0.54

a Chi-squared; Fisher's exact test.

b Asthma exacerbations were measured by parent report of the child requiring oral steroids, having emergency department visit, or having hospitalization related to asthma in the past 12 months.

## Discussion

This observational study is among the first to describe quick-relief inhaler storage and use across children's daily settings, which is important as the ability to respond efficiently to respiratory symptoms is a key component to asthma management Further, this study examines their association with asthma control, providing insights about fostering supportive asthma environments and effective asthma care among youth experiencing acute symptoms.

We observed that a majority of 10–17 year old participants self-carry and use inhalers at home and at school, similar to a past study on inhaler self-carry among younger children (8–13 years) (4). While promising, these results highlight that ∼40% of children did not independently carry and/or use their inhalers. Children should be encouraged to develop the skills for independent inhaler storage/use across the various settings where they live, learn, and play, particularly as they become increasingly independent in day-to-day activities. Lack of independence may contribute to delayed care, as research shows that inhaler storage in the office at school can result in children postponing communication of their symptoms (5). Such delays may affect the management of respiratory events and resulting asthma outcomes.

Literature shows that children can learn to self-manage their asthma as young as 7 years old (6), and independence increases with age (7, 8). Our results align in part with the literature, showing greater independent inhaler storage at school among the older group (14–17 years) than the younger group (10–13 years), although no differences were seen by age in terms of inhaler storage at home as well as use inhaler use at home and school. Notably, factors at various levels can influence preparedness for self-carry/use, including the child's knowledge of asthma and inhalers (individual) (4), parent familiarity with school practices (interpersonal) (8), school asthma policy (organization), and financial costs of medications (system). Notably, caregivers report challenges with transitioning chronic condition management from caregiver shared-management to child self-management (9). Thus, healthcare professionals and school nurses must work closely with families to support such transitions in an age and developmentally appropriate way to achieve self-management. Efforts may include discussing inhaler self-carry and use as part of asthma education by primary care clinicians and school nurses to promote independence. Such practices across both health and school systems can help foster asthma self-management to support improved outcomes (3, 4).

Our study found that behaviors like storing quick-relief inhalers independently at home and school align with independent inhaler use in each setting. In addition, children who independently used their inhalers at home did the same at school. Thus, promoting independent inhaler use at home may boost confidence and self-management skills that translate to other settings. However, independent storage of quick-relief inhalers was not correlated across settings, potentially reflecting the complexity of inhaler storage as a child moves across their daily settings. While inhaler use relies more on the child and if needed one adult for support, multiple factors may affect the inhaler storage. For example, a child's ability to self-carry their inhaler at school may be affected by the presence of school nurse, staff knowledge about asthma, existence of asthma policies, severity of a student's asthma, and concern about other students’ medication access. Despite existing laws in all 50 states requiring schools to permit self-carry, the implementation and enforcement of many such policies are left to school districts and/or individual schools, leading to inconsistent implementation, a cited barrier to inhaler self-carry (4). The variation in inhaler storage across settings reinforces the need for comprehensive, collaborative asthma care that requires integrated policy and partnership among individuals across settings (e.g., school nurses, administrators, teachers).

In our study, we examined the storage and use of quick-relief inhalers specifically and did not observe any associations between asthma control or asthma exacerbations and independent inhaler storage or use at home or school. Asthma control may not have been affected as short-acting beta agonists do not impact the underlying pathophysiology that drives disease control. Also, inhaler storage and use practices may differ between quick-relief and controller medications. Further, our results may reflect variations in quick-relief inhaler storage and use among our population (e.g., wrong technique, poor timing, not using spacers). It is possible that children with well-controlled asthma may be more independent with their inhalers; at the same time, children with poorly controlled asthma may be more likely to independently store and use their inhalers as they need to use the medication more often. While a child may demonstrate independence with their inhaler, if they are not using the inhaler properly, the child may experience poorly controlled asthma or acute asthma exacerbations. In fact, literature highlights a discrepancy between children's self- and parent-reported readiness to self-carry with their objective readiness (7, 8), emphasizing the value of incorporating objective assessments when counseling about transitions from shared management to child self-management. In addition, our results may be explained by other factors influencing asthma, such as environmental triggers. Independent inhaler storage and use cannot mitigate symptoms in an environment fraught with asthma triggers, for example, mold or smoke, which contribute to asthma exacerbations and are shown to worsen asthma morbidity (10). Future research should explore the relationship between inhaler storage/use patterns and asthma outcomes to help identify optimal approaches to support self-management as children develop.

For limitations, our study assessed quick-relief inhaler storage/use patterns in a population with high rates of well-controlled asthma who resides in an urban community with high asthma prevalence, potentially limiting its generalizability. In addition, given the small sample size, we did not examine findings by demographic characteristics. We also specifically focused on storage and use of quick-relief inhalers (short acting beta agonists), which may be distinct from practices with controller medications. Further, the cross-sectional design does not allow us to establish causality, and our results are subject to response and recall bias given a survey was used to examine inhaler storage/use patterns. Notably, this study took place between 2020 and 2021, which aligned with the peak of the COVID-19 pandemic, which may have affected reports of inhaler storage and use, given varying practices in terms of in-person learning and school-based asthma care during the pandemic. To help overcome these limitations, future research can assess inhaler storage and use objectively as well as investigate more in-depth factors that may affect inhaler storage and use (e.g., age, sex, race/ethnicity, urban/rural setting, disease severity).

Overall, the patterns observed regarding independent quick-relief inhaler use and storage at home and school underscore the importance of continued, open dialogue between healthcare professionals, caregivers, and children about developmentally appropriate asthma self-management, particularly in case of symptoms. Importantly, asthma care is multi-faceted; thus, promoting positive asthma outcomes requires combining such efforts with further work in reducing environmental triggers and coordinating caregiver, school nurses, and health care professionals across various settings. By supporting self-care, in combination with comprehensive asthma education, caregivers and clinicians in school and health systems can help empower children to effectively self-manage their symptoms and improve asthma outcomes.

## Data Availability

The raw data supporting the conclusions of this article will be made available by the authors, without undue reservation.
